# Determinants on urinary excretion of oxalate and other key factors related to urolithiasis among patients with chronic kidney disease: a single center study

**DOI:** 10.1007/s00240-023-01458-y

**Published:** 2023-06-14

**Authors:** Daorina Bao, Huimin Zhang, Jinwei Wang, Yu Wang, Suxia Wang, Ming-hui Zhao

**Affiliations:** 1 Renal Division, Department of Medicine, Peking University First Hospital, No. 8 Xishiku St., Xicheng District, Beijing, 100034 China; 2 Institute of Nephrology, Peking University, Beijing, 100034 China; 3 Key Laboratory of Renal Disease, National Health and Family Planning Commission of the People’s Republic of China, Beijing, 100034 China; 4 Key Laboratory of Chronic Kidney Disease Prevention and Treatment, Ministry of Education, Beijing, 100034 China; 5 Research Units of Diagnosis and Treatment of Immune-Mediated Kidney Diseases, Chinese Academy of Medical Sciences, Beijing, China; 6 Laboratory of Electron Microscopy, Pathological Centre, Peking University First Hospital, Beijing, 100034 China; 7 Peking-Tsinghuric Center for Life Sciences, Beijing, 100871 China

**Keywords:** Urolithiasis, Chronic kidney disease, Oxalate, eGFR, Urinary protein excretion, Pathological change

## Abstract

**Purpose:**

Urolithiasis is a known risk factor for chronic kidney disease (CKD). However, how CKD might affect the risk of incidence of urolithiasis is not widely studied.

**Methods:**

Urinary excretion of oxalate as well as other key factors related to urolithiasis was analyzed in a single center study of 572 patients with biopsy-proven kidney disease.

**Results:**

The mean age of the cohort was 44.9 years and 60% were males. The mean eGFR was 65.9 ml/min/1.73 m^2^. Median urinary excretion of oxalate was 14.7 (10.4–19.1) mg/24-h and associated with current urolithiasis (OR 12.744, 95% CI: 1.564–103.873 *per* one logarithm transformed unit of urinary oxalate excretion). Oxalate excretion was not associated with eGFR and urinary protein excretion. Oxalate excretion was higher in patients with ischemia nephropathy as compared with patients with glomerular nephropathy and tubulointerstitial nephropathy (16.4 vs 14.8 vs 12.0 mg, *p* = 0.018). And ischemia nephropathy (*p* = 0.027) was associated with urinary oxalate excretion on adjusted linear regression analysis. Urinary excretion of calcium and uric acid was correlated with eGFR and urinary protein excretion (all *p* < 0.001), with ischemia nephropathy and tubulointerstitial nephropathy associated with uric acid excretion (both *p* < 0.01) as well. Citrate excretion was correlated with eGFR (*p* < 0.001) on adjusted linear regression.

**Conclusion:**

Excretion of oxalate and other key factors related to urolithiasis was differentially associated with eGFR, urinary protein, and pathological changes in CKD patients. The influence of these intrinsic traits of the underlining kidney disease should be considered when evaluating urolithiasis risk in patients with CKD.

**Supplementary Information:**

The online version contains supplementary material available at 10.1007/s00240-023-01458-y.

## Introduction

Chronic kidney disease (CKD) is a widely recognized public health concern, with an estimated prevalence of 10.8% in China [1]. The spectrum of CKD etiology in China has changed dramatically in recent years. According to the China Kidney Disease Network reports, obstructive nephropathy has risen to the third most common cause of CKD in China with urolithiasis as the major contributor [2]. Previous studies have shown that urolithiasis is a risk factor for the development of CKD and end stage kidney disease (ESKD) [3–5]. However, how CKD might affect the risk of incidence of urolithiasis is not widely studied. There is a paucity of data evaluating the impact of CKD on urinary mineral excretion.

Calcium oxalate is the most common type of urolithiasis [6]. Circulating oxalate is freely filtered at the glomerulus, reabsorbed and secreted by the tubules. Even a small increase in urinary oxalate has a significant impact on development of an incidental kidney stone. The excretion of oxalate has been evaluated extensively in patients with urolithiasis [7–9], but few data has ever reported in patients with CKD. Recently, the researchers of Chronic Renal Insufficiency Cohort (CRIC) study observed an inverse correlation of 24-h urinary oxalate excretion with eGFR [10], which was different from the results of most studies of patients with urolithiasis [7–9]. This discrepancy suggested that the regulation of urinary mineral excretion might be more complex in patients with CKD. In the present study, we measured urinary excretion of oxalate and other key factors related to urolithiasis as well in a cohort of patients with biopsy-proven kidney disease, hoping to add more information to this field.

## Methods

### Patient cohort

Our study was approved by the ethics board of Peking University First Hospital (2021[0236]) and performed in accordance with the Declaration of Helsinki. Adult patients who were admitted from January 2021 to May 2022 were prospectively enrolled. Exclusion criteria included inability to provide consent, anuria/oliguria upon presentation, pregnancy, liver cirrhosis, New York Heart Association class III to IV congestive heart failure, recent chemotherapy for malignancy including hematologic tumors, hereditary kidney disease, kidney transplantation, or prior treatment with dialysis for at least 1 month. Those who did not receive renal biopsy were also excluded. Incomplete urine collection (urinary creatinine < 800 mg/day for male and < 600 mg/day for female, respectively) was further excluded. Given that calcium supplementation might affect oxalate absorption from gut, those who were taking calcium at the time of urine collection were further excluded. The flowchart of patient selection was shown in Supplementary Fig. 1. All participants provided written informed consent and all data were de-identified.

### Data collection

Demographic (age, sex), medical history (hypertension, diabetes, gout, urolithiasis, coronary heart disease, dyslipidemia), and anthropometric measure (weight, height) were collected from electronic medical charts. Laboratory data of serum and urinalysis and current medication at the time of renal biopsy were also collected.

Patients were categorized according to eGFR calculated by the CKD-EPI equation as: ≥ 90 ml/min/1.73 m^2^, 60–89 ml/min/1.73 m^2^, 30–59 ml/min/1.73 m^2^, 15–29 ml/min/1.73 m^2^, and < 15 ml/min/1.73 m^2^, respectively. Serum calcium level (mmol/l) was corrected by the following equation: measured serum calcium level (mmol/l) + 0.02 × (40-serum albumin level) (g/l).

### Sample collection, handling and measurement

Participants were provided a 24-h urine collection container the morning before renal biopsy and started to collect urine sample after voiding actively at 7 a.m. And then, participants urinated actively at 7 a.m. the second morning to end the collection with the last urine sample being collected in the container. The total urine volume was recorded and aliquots were taken after through mixing. Then, the urine samples were centrifuged with 4500r per minute for 15 min at 4 °C as soon as possible. Supernatant was sub-aliquoted and kept at − 20 °C for further measurement. Urine oxalate and citrate measurement was performed by Aquion RFIC (Thermo Scientific, USA). In brief, 1 ml of supernatant was acidified with 10 μl 12N hydrochloric acid and diluted 1:5–1:10 with purified water (Milli-Q Synthesis, USA). Then, 5 ml diluted samples were de-proteinized by filtration through Dionex OnGuard II RP 1 cc Cartridge (Thermo Fisher Scientific, USA) and 700 μl processed samples were used for final measurement. Concentrations of potassium, sodium, chlorine, calcium, phosphate, magnesium, creatinine and uric acid were measured using the standard chemical colorimetric method. The 24-h excretion was calculated by multiplying the concentration by the urinary volume. Standardized estimates of the ion active products of calcium oxalate (AP_CaOx_ index) and calcium phosphate (AP_CaP_ index) was calculated according to the formulas given below [11, 12]. In these calculations, 24-h urine ions were expressed in mmol and the volume in liters.

$${\text{AP}}_{{{\text{CaOx}}}} \,{\text{index}} = {1}.{9} \times {\text{calcium}}^{{0.{84}}} \times {\text{oxalate}}/{\text{citrate}}^{{0.{22}}} /{\text{magnesium}}^{{0.{12}}} /{\text{volume}}^{{{1}.0{3}}}$$

$${\text{AP}}_{{{\text{CaP}}}} \;{\text{index}} = {2}.{7} \times {1}0^{{{-}{3}}} \times {\text{calcium}}^{{{1}.0{7}}} \times {\text{phosphate}}^{{0.{7}0}} \times ({\text{pH}} - {4}.{5})^{{{6}.{8}}} /{\text{citrate}}^{{0.{2}0}} /{\text{volume}}^{{{1}.{31}}}$$

### Statistical analysis

Continuous variables were expressed as means (SDs) or median (25th, 75th percentiles) and compared with parametric (Students’ t-test or analysis of variance (ANOVA)) or nonparametric tests (Mann–Whitney U test, or Kruskal–Wallis H test), as appropriate. Categorical variables were expressed as percentages and analyzed with Chi-square test. Logistic regression analysis by forward likelihood ratio method was used to analyze the association of patient characteristics with urolithiasis (entry criterion *p* < 0.05). Odds ratios (ORs) and 95% confidence intervals (CIs) were estimated. General linear regression model was performed to determine the variables that were associated with oxalate and other key urinary factors with the inclusion of variate with *p* < 0.05 by univariate analysis. A two-sided *p*-value of $<$ <0.05 was considered statistically significant. All analyses were performed using SPSS software, version 27.

## Results

Totally, 572 patients were enrolled with a mean age of 44.9 (14.1) years and 343 (60.0%) were males. The mean eGFR was 65.9 (32.7) ml/min/1.73 m^2^. The median urinary protein excretion was 1.9 (0.8, 4.1) g/d. According to the major pathological finding on renal biopsy, the majority of patients (505/572, 88.3%) were with glomerular nephropathy (GN). There were another 33 (5.8%) and 34 (5.9%) patients found with tubulointerstitial nephropathy (TIN) and ischemia nephropathy as primary pathological diagnosis, respectively. Baseline characteristics of participants according to eGFR categories are given in Table 1. No significant difference in the history of urolithiasis and current urolithiasis was found among patients with different eGFR categories.

**Table 1:** Demographic and clinical characteristics of the study cohort according to eGFR categories

	All participants	eGFR ≥ 90	eGFR 60–89	eGFR 30–59	eGFR 15–29	eGFR < 15	*p*
*No. (%)*	572	171 (29.9)	141 (24.7)	160 (28.0)	71 (12.4)	29 (5.1)	/
Age, years	44.9 (14.1)	39.5 (12.5)	45.9 (14.0)	46.6 (14.1)	49.3 (14.0)	52.1 (14.4)	< 0.001
Male, no. (%)	343 (60.0)	91 (53.2)	83 (58.9)	104 (65.0)	40 (56.3)	25 (86.2)	0.008
BMI, kg/m^2^	24.9 (3.9)	24.7 (4.1)	24.9 (3.8)	25.0 (3.9)	25.3 (4.2)	25.3 (3.6)	0.782
*Past medical history, no. (%)*							
Hypertension	319 (55.8)	58 (33.9)	71 (50.4)	111 (69.4)	57(80.3)	22 (75.9)	< 0.001
Diabetes	109 (19.1)	11 (6.4)	23 (16.3)	40 (25.0)	23 (32.4)	12 (41.4)	< 0.001
Gout	28 (4.9)	2 (1.2)	7 (5.0)	13 (8.1)	5 (7.0)	1 (3.4)	0.049
Urolithiasis	16 (2.8)	4 (2.3)	4 (2.8)	5 (3.1)	2 (2.8)	1 (3.4)	0.993
*Medications, no. (%)*							
Diuretic	115 (20.1)	32 (18.7)	28 (19.9)	28 (17.5)	17 (23.9)	10 (34.5)	0.261
RASI	357 (62.4)	116 (67.8)	92 (65.2)	100 (62.5)	36 (50.7)	13 (44.8)	0.031
Statin	179 (31.3)	52 (30.4)	41 (29.1)	48 (30.0)	27 (38.0)	11 (37.9)	0.627
Glucortical steroid/immunosuppression agents	26 (4.5)	5 (2.9)	0 (0)	10 (6.3)	7 (9.9)	4 (13.8)	0.001
Sodium bicarbonate	92 (16.1)	9 (5.3)	11 (7.8)	35 (21.9)	23 (32.4)	14 (48.3)	< 0.001
*Blood parameters*							
Scr, μmol/l	107.0 (78.7, 163.9)	69.9 (60.2, 79.8)	95.9 (84.5, 105.9)	144.9 (128.8, 168.6)	238.8 (201.4, 281.0)	437.2 (381.4, 525.6)	< 0.001
eGFR, ml/min/1.73 m^2^	65.9 (32.7)	105.4 (9.8)	74.6 (9.4)	44.4 (8.5)	23.4 (4.3)	11.0 (2.6)	< 0.001
Urinary protein, g/24 h	1.9 (0.8, 4.1)	1.7 (0.7, 3.5)	1.5 (0.7, 3.2)	1.7 (0.7, 4.2)	2.0 (1.1, 4.6)	3.4 (1.0, 7.2)	0.019
Serum albumin, g/l	36.1 (7.2)	34.5 (7.7)	36.5 (7.3)	37.5 (6.8)	36.9 (6.6)	34.5 (5.9)	0.002
Hemoglobin, g/l	129.7 (22.0)	140.7 (16.6)	135.6 (18.4)	126.4 (20.8)	112.5 (19.3)	97.6 (18.3)	< 0.001
Uri acid, mg/dl	6.8 (1.7)	6.1 (1.5)	6.7 (1.5)	7.2 (1.8)	7.3 (2.0)	7.1 (1.8)	< 0.001
Calcium, mmol/l	2.39 (0.09)	2.39 (0.08)	2.41 (0.08)	2.39 (0.09)	2.37 (0.09)	2.32 (0.10)	< 0.001
Phosphate, mmol/l	1.15 (0.23)	1.12 (0.18)	1.09 (0.19)	1.13 (0.22)	1.25 (0.25)	1.45 (0.39)	< 0.001
CO_2_, mmol/l	25.9 (2.9)	27.0 (2.3)	27.0 (2.3)	25.7 (2.6)	24.0 (3.1)	21.1 (2.4)	< 0.001
*Current urolithiasis*	20 (3.5)	5 (2.9)	6 (4.3)	5 (3.1)	4 (5.6)	0 (0)	0.647
*Pathological type, no. (%)*							< 0.001
GN	505 (88.3)	169 (98.9)	131 (92.9)	133 (83.1)	52 (73.2)	20 (69.0)	
TIN	33 (5.8)	0 (0)	2 (1.4)	14 (8.8)	10 (14.1)	7 (24.1)	
Ischemia nephropathy	34 (5.9)	2 (1.2)	8 (5.7)	13 (8.1)	9 (12.7)	2 (6.9)	
*Urine parameters*							
Volume, ml/24 h	2073 (664)	2064 (660)	2013 (635)	2098 (648)	2194 (788)	1994 (573)	0.383
pH	6.1 (0.5)	6.3 (0.5)	6.1 (0.5)	6.0 (0.5)	5.9 (0.5)	6.0 (0.6)	< 0.001
Calcium, mmol/24 h	1.4 (0.6, 2.5)	2.4 (1.4, 3.6)	1.5 (0.8, 2.7)	0.9 (0.5, 1.7)	0.8 (0.5, 1.4)	0.7 (0.5, 1.2)	< 0.001
Phosphate, mmol/24 h	15.8 (11.9, 20.9)	16.4 (12.7, 20.9)	15.8 (11.9, 21.0)	15.5 (11.9, 21.1)	16.0 (11.7, 20.9)	11.8 (9.6, 16.2)	0.118
Magnesium, mmol/24 h	3.3 (1.4)	3.2 (1.4)	3.3 (1.5)	3.4 (1.4)	3.2 (1.2)	3.1 (1.3)	0.677
Oxalate, mg/24 h	14.7 (10.4, 19.1)	14.9 (10.7, 19.2)	14.6 (9.9, 19.8)	14.6 (10.3, 18.1)	14.2 (10.3, 19.4)	15.4 (12.2, 19.6)	0.815
Citrate, mg/24 h	181 (87, 297)	238 (111, 414)	213 (109, 346)	148 (73, 255)	128 (68, 203)	101 (48, 202)	< 0.001
Uric acid, mg/24 h	314 (214, 450)	393 (272, 475)	299 (218, 451)	282 (184, 390)	284 (169, 443)	246 (204, 355)	< 0.001
Creatinine, mg/24 h	1199 (950, 1568)	1252 (989, 1730)	1230 (951, 1583)	1159 (955, 1526)	1162 (932, 1478)	1071 (907, 1289)	0.118
AP_CaOx_	0.17 (0.09, 0.30)	0.26 (0.15, 0.47)	0.17 (0.10, 0.33)	0.13 (0.08, 0.23)	0.10 (0.07, 0.22)	0.13 (0.08, 0.25)	< 0.001
AP_CaP_	0.22 (0.03, 1.04)	0.72 (0.16, 3.15)	0.28 (0.04, 1.17)	0.11 (0.01, 0.36)	0.06 (0.01, 0.23)	0.06 (0.01, 0.33)	< 0.001

The normal range of urinary excretion for protein: 0–150 mg/24 h, for calcium: 2.5–7.5 mmol/24 h, for phosphate: 9.7–42 mmol/24 h, for magnesium: 0.98–10.49 mmo/24 h, for oxalate: < 40 mg/24 h, for citrate: > 320 mg/24 h, for uric acid: 250–750 mg/24 h, respectively

*BMI* Body Mass Index, *eGFR* estimated glomerular filtration rate, ml/min/1.73 m^2^, *GN* glomerular nephropathy, *TIN* tubulointerstitial nephropathy

**Fig. 1:**
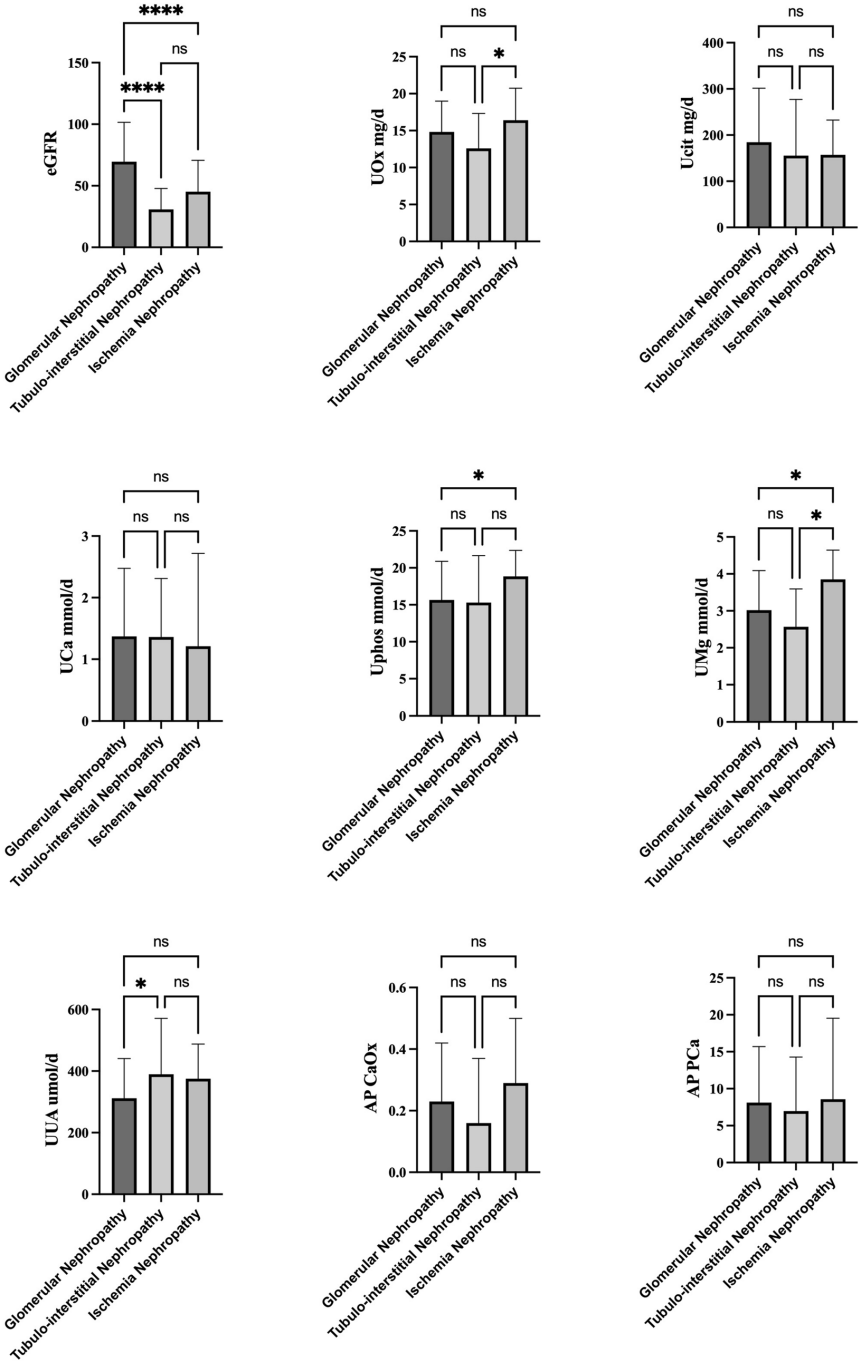
Urinary excretion of minerals and active product of calcium oxalate and calcium phosphate by pathological types

Urinary excretion of oxalate was 14.7 (10.4, 19.1) mg/d of the cohort. Patients with current urolithiasis had higher urinary oxalate excretion than those without (19.7 vs 14.6 mg, *p* = 0.002). Logistic analysis showed that denary logarithm urinary oxalate excretion (OR 12.744, 95% CI: 1.564–103.873) was independently associated with current urolithiasis (Table 2). No significant difference of urinary oxalate excretion was found among different categories of eGFR (Table 1). Median urinary oxalate excretion was 16.4 mg (12.0, 20.7) in patients with ischemia nephropathy, which was higher than 14.8 mg (10.6, 18.9) in GN and 12.0 mg (7.3, 17.9) in TIN (*p* = 0.018), respectively (Fig. 1). Urinary protein excretion was associated with urinary oxalate excretion in un-adjusted analysis, which lost significance after multivariable adjustments (Table 3). The variables associated with adjusted oxalate excretion were ischemia nephropathy (*p* = 0.027), current urolithiasis (*p* = 0.046), higher BMI (*p* = 0.001), and urine pH (*p* = 0.001) in adjusted linear regression model (Table 3).

**Table 2:** Univariable and multivariable logistic analysis of current urolithiasis

	Univariable			Multivariable adjusted		
	OR	95%CI	*p* value	OR	95%CI	*p* value
Age (per 5 years)	0.632	0.239–1.670	0.355	/	/	/
Female vs male	1.063	0.909–1.243	0.447	/	/	/
*Diabetes*	2.977	1.186–7.472	0.020	2.643	1.003–6.966	0.049
Hypertension	1.888	0.716–4.990	0.199	/	/	/
*Gout*	3.720	1.023–13.531	0.046	/	/	/
*Past urolithiasis*	11.250	3.269–38.720	< 0.001	7.823	1.884–32.483	0.005
BMI (per 1 unit)	1.010	0.903–1.128	0.866	/	/	/
eGFR (per 10 ml/min/1.73 m^2^)	0.998	0.984–1.011	0.742	/	/	/
Uric acid	0.915	0.705–1.187	0.504	/	/	/
Calcium	3.882	0.027–563.811	0.593	/	/	/
Phosphate	1.032	0.154–6.927	0.975	/	/	/
CO_2_	0.941	0.811–1.092	0.424	/	/	/
urine pH	0.860	0.369–2.006	0.727	/	/	/
*Urinary protein (per g/24 h)*	1.103	1.004–1.212	0.041	/	/	/
Urine volume (L/24 h)	0.873	0.435–1.752	0.703	/	/	/
Ucal	1.264	0.399–4.009	0.690	/	/	/
Uphos	1.231	0.113–13.385	0.864	/	/	/
U_UA_	1.000	0.998–1.003	0.879	/	/	/
*Denary logarithm urine oxalate*	16.463	2.115–128.146	0.007	12.744	1.564–103.873	0.017
Ucit	1.002	1.000–1.004	0.053	/	/	/
RASI	1.161	0.376–3.584	0.795	/	/	/
Diuretic	1.742	0.654–4.637	0.267	/	/	/
*Pathological type*						
GN	1.000					
TIN	0.897	0.116–6.956	0.917	/	/	/
Ischemia nephropathy	1.794	0.397–8.107	0.447	/	/	/

*BMI* Body Mass Index, *eGFR* estimated glomerular filtration rate, *GN* glomerular nephropathy, *TIN* tubulointerstitial nephropathy, *Ucal* urine calcium, *Uphos* urine phosphate, *U*_*UA*_ urine uric acid, *Ucit* urine citrate, *RASI* renin-angiotensin-system inhibitor

**Table 3:** Factors associated with 24-h urinary excretion of oxalate and other key factors related to urolithiasis by linear regression analysis

	Oxalate, mg/day			Calcium, mmol/day			Phosphate, mmol/day			Magnesium, mmol/day			Uric acid, mg/day			Citrate, mg/day		
	Bi (B)	MV (B)	*p*	Bi (B)	MV (B)	*p*	Bi (B)	MV (B)	*p*	Bi (B)	MV (B)	*p*	Bi (B)	MV (B)	*p*	Bi (B)	MV (B)	*p*
Age (per 5 years)	− 0.036			− 0.022			− 0.042			0.015			− 0.045			0.033		
Female vs male	− 0.036			− 0.108**	− 0.165	< 0.001	− 0.361***	− 0.317	< 0.001	− 0.255***	− 0.204	< 0.001	− 0.045			− 0.028		
Diabetes	0.098*			− 0.086*			0.118**			0.189***	0.161	< 0.001	0.01			0.122**	0.187	< 0.001
Hypertension	0.061			− 0.103*			0.075			0.095**			− 0.057			− 0.05		
Past urolithiasis	0.014			0.111**	0.082	0.028	0.077			0.045			0.011			− 0.02		
Gout	− 0.022			0.017			0.081			0.109***			− 0.05			− 0.03		
BMI (per 1 unit)	0.166***	0.149	0.001	0.071			0.290***	0.222	< 0.001	0.153***			0.086**			0.146***	0.174	< 0.001
eGFR (per 10 ml/min/1.73 m^2^)	0.049			0.399***	0.411	< 0.001	0.08			− 0.014			0.203***	0.267	< 0.001	0.294***	0.251	< 0.001
Uric acid	0.031			− 0.132**	− 0.097	0.019	0.181***			0.142***			− 0.072			− 0.144***	− 0.126	0.002
Serum calcium	− 0.043			0.167***	0.136	0.001	− 0.002			− 0.033			− 0.02			0.198***	0.144	< 0.001
Serum phosphate	0.035			− 0.216***			0.002			0.002			− 0.079			− 0.067		
Bicarbonate	− 0.041			0.155***			0.034			0.061			0.086**			0.251***	0.126	0.004
Urine pH	0.105*	0.139	0.001	0.090**			− 0.053			− 0.036			0.222***	0.127	0.004	0.084**		
Urinary protein (per g/24 h)	0.096*			− 0.208***	− 0.148	< 0.001	0.091*			0.059			0.166***	0.204	< 0.001	0.006		
Diuretic	0.083*			− 0.103**			0.04			0.003			0.038			− 0.029		
Urolithiasis	0.100*	0.083	0.046	0.06			0.015			− 0.004			0.006			0.087*		
TIN vs GN	− 0.085*			0.059			− 0.020			− 0.051			0.095*	0.205	< 0.001	− 0.017		
Ischemia nephropathy vs GN	0.096*	0.094	0.027	− 0.003			0.061			0.092*			0.026	0.117	0.005	− 0.075		

*Bi* bivariate association, *MV* multivariate association; *β* beta estimate, standardized, *BMI* Body Mass Index, *eGFR* estimated glomerular filtration rate, *GN* glomerular nephropathy, *TIN* tubulointerstitial nephropathy

*p < 0.05; **p < 0.01; ***p < 0.001

There was a trend of decrease of urinary calcium, citrate and uric acid excretion with the declination of eGFR, accompanied with decrease of both AP_CaOx_ index and AP_CaP_ index (Table 1). Adjusted linear regression analysis showed that eGFR was correlated positively with urine calcium, uric acid, and citrate excretion (Table 3). Urine protein was found correlated with urine calcium inversely and uric acid excretion positively in adjusted linear regression model (both *p* < 0.001) (Table 3). Difference in phosphate, magnesium and uric acid excretion was observed among different pathological types (Fig. 1). And ischemia nephropathy and TIN was found associated with uric acid excretion in adjusted linear regression model (both *p* < 0.01) (Table 3).

## Discussion

Chronic kidney disease is a worldwide public health concern. So does kidney stones [13–15]. Patients with urolithiasis are known at increased risk for CKD and sustained reduction in GFR [3–5]. But, whether vice versa is true has not been well studied. In the present study, we enrolled a large number of patients with various biopsy-proven kidney diseases to evaluate how urinary excretion of minerals related to urolithiasis was affected by CKD.

Approximately 70–80% of the kidney stones contain oxalate. Even small increase in urinary oxalate could increase the risk of developing kidney stones [6]. In the present study, urinary oxalate excretion was independently associated with current urolithiasis, supporting its key role as a risk factor for stone formation in patients with CKD also. Besides, urinary oxalate excretion has been demonstrated to cause renal parenchymal disease [16–19] and associated with progression of CKD [10]. Despite the importance of oxalate to both urolithiasis and CKD, there are few data about the characteristics of oxalate excretion in patients with CKD. The only data from CRIC study showed an inverse correlation between urinary oxalate excretion and eGFR in patients with CKD stage 2–4 [10]. This result was different to those from studies of patients with urolithiasis solely, most of which showed paralleled decrease of urine oxalate excretion with GFR declination [7, 9]. We examined the urine oxalate excretion in a cohort of Chinese patients with biopsy-proven kidney disease in the present study. The level of urinary oxalate excretion was relatively stable among different eGFR categories without significant association observed between eGFR and oxalate excretion. The internal validity of our result is supported by consistency with the reported literature on the associations of oxalate excretion with BMI and current urolithiasis [20]. Oxalate is filtered freely via glomeruli and undergoes passive absorption and active secretion in the proximal tubules by transporters from the solute carrier family, resulting net excretion. These procedures might be affected differentially by the underlining kidney disease, which we thought might partly explain the difference observed between patients with kidney disease and urolithiasis solely and among patients with different underlining kidney diseases. For instance, the participants in CRIC had a median proteinuria of 0.17 g/24-h. In contrast, the majority of our patients presented with severe proteinuria with biopsy-proven glomerular nephropathy. The difference of proteinuria level suggested a different constitutions of underlining kidney diseases of the two studies. The difference of oxalate excretion among different pathological types found in our study gave some support to our speculation. But we acknowledged that there was indeed an overlap for oxalate excretion among the three pathological groups. Therefore, the true role of different pathological types on oxalate excretion needed to be studied further. In addition, metabolic or intestinal alterations may influence urinary oxalate excretion, which might be affected differently by underlining kidney diseases.

The formation of kidney stone is a complex and multistep process that includes urinary supersaturation, crystal nucleation, growth and aggregation [21]. Urinary supersaturation is the driving force behind crystal formation in the kidneys than individual component concentration. We found that urine components both favor for crystallization as calcium and uric acid and protective against crystallization as citrate were all associated positively with eGFR. Accordingly, a decrease in AP_CaOx_ and AP_CaP_ with decreasing eGFR was observed. This result suggests that the stone risk might decrease as CKD progresses. However, the alterations induced by underlining kidney disease on other processes involved in stone formation should also be taken into account. For instance, the transit time for urine to flow across the kidney which may affect the nucleation and growth of crystals to become large enough to be trapped in the kidney [22] might be altered with eGFR change. And, the change of urinary tract epithelium status under CKD may affect the adhesion of crystals to epithelium either. Therefore, more epidemiological studies are needed to definitely answer the question of how the risk of urolithiasis incidence changed with declination of eGFR in patients with established CKD.

There are several limitations of our study. As a study carried out in a tertiary care center, our cohort was subject to certain selection bias and likely comprised higher risk patients with kidney disease. Therefore, it is not appropriate to extrapolate our results directly to the more generalized CKD populations. Only one sample of 24-h urine of each patient was collected and the impact of diet on patients’ 24-h urine parameters independent of renal function was not assessed.

In conclusion, we evaluated the urinary mineral excretion related to urolithiasis in a large cohort of patients with biopsy-proven kidney diseases. Our findings showed different correlations of eGFR, urine protein, and pathological change with oxalate as well as other key urinary factors excretion. The influence of these intrinsic traits of underlining kidney disease should be taken into account when evaluating urolithiasis risk in patients with CKD.

### Supplementary Information

Below is the link to the electronic supplementary material.

Flowchart of patient selection (JPG 179 KB)

## Data Availability

The data underlying this article will be shared on reasonable request to the corresponding author.
