# Supplementary figures and images for: Determinants on urinary excretion of oxalate and other key factors related to urolithiasis among patients with chronic kidney disease: a single center study
Source: Urolithiasis. 2023 Jun 14;51(1):88. doi: 10.1007/s00240-023-01458-y (PMC10266999; doi:10.1007/s00240-023-01458-y)

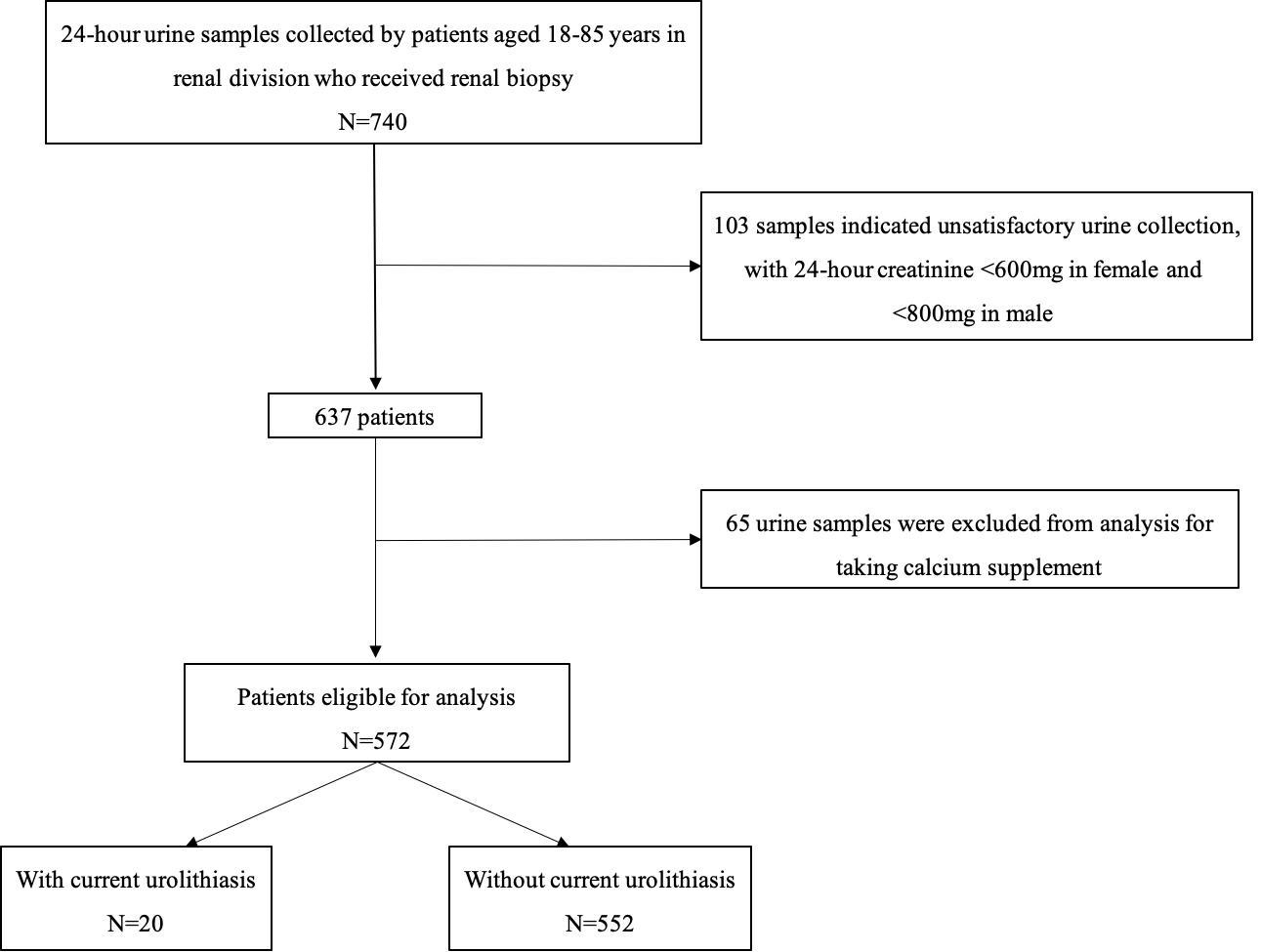

Supplement: Supplementary file 1 — Flowchart of patient selection (JPG 179 KB) [file 240_2023_1458_MOESM1_ESM.jpg]
